# Impact of physiological strain on lung epithelial cells by exposure to aerosolised quartz silica in a perfused bioreactor

**DOI:** 10.3389/fbioe.2026.1846147

**Published:** 2026-06-15

**Authors:** Ludovica Cacopardo, Nathalie Jung, Nicole Guazzelli, Roberta Nossa, Sandeep Keshavan, Mira Witzig, Alain Rohrbasser, Mauro Sousa de Almeida, Alke Petri-Fink, Arti Ahluwalia, Barbara Rothen-Rutishauser

**Affiliations:** 1 Department of Information Engineering and Research Centre E. Piaggio, University of Pisa, Pisa, Italy; 2 BioNanomaterials Group, Adolphe Merkle Institute, University of Fribourg, Fribourg, Switzerland; 3 PBPK M&S Consulting Department, RANDSTAD DIGITAL ITALY S.r.l., Milano, Italy; 4 ItalyScientific Institute, IRCCS Eugenio Medea, Bosisio Parini (LC), Bosisio Parini, Italy

**Keywords:** aerosol exposure, air–liquid interface, bioreactor, crystalline silica (DQ12), cyclic strain, lung epithelial cells

## Abstract

Understanding how inhaled particles that can reach the deep lung induce adverse effects requires *in vitro* platforms that reflect key exposure and mechanical features of the alveolar environment. Here, we engineered a dynamic air–liquid interface bioreactor integrating physiological cyclic strain, basal perfusion, and apical aerosol delivery. Cyclic breathing-like deformation of an elastic membrane is driven by pressure-regulated airflow, while real-time aerosol deposition is quantified using an integrated quartz crystal microbalance, enabling precise control of delivered dose. Human alveolar epithelial cells (A549) cultured under breathing-like conditions exhibited moderate, adaptive changes in morphology, cell density, and gene expression, indicative of a low-stress mechanoadaptive state. Using this platform, cells were exposed to aerosolised crystalline quartz (DQ_12_) at a sub-cytotoxic dose, representative of occupational exposure. Particle deposition under dynamic conditions induced distinct transcriptional shifts in A549 cells, particularly in inflammatory and adhesion-related genes, without triggering broader stress or mucin-associated responses. Together, these results demonstrate how coupling physiologically-relevant mechanical actuation with quantitative aerosol exposure provides a robust physiological platform for investigating particle–lung epithelial interactions under realistic human exposure scenarios.

## Introduction

Air pollution is one of the leading global health risks, with approximately 6.7 million premature deaths annually and 99% of the world’s population living in areas that exceed the World Health Organisation’s (WHO) air quality guidelines (WHO, 2024). Of particular concern among air pollutants are fine particles with aerodynamic diameters below 2.5 µm, also referred to as PM2.5, and ultrafine particles (PM0.1) (Schraufnagel, 2020). These particles originate from combustion processes, industrial emissions, and mechanical abrasion, and are strongly associated with respiratory and cardiovascular diseases due to their ability to induce oxidative stress and inflammation in lung tissue (Schraufnagel, 2020; Riffault et al., 2015). Occupational exposure to airborne particles, which often entails lower concentrations and particles with distinct physicochemical properties, poses a significant health risk (Nishida and Yatera, 2022). Protecting workers from inhalation hazards is not only a matter of personal protective equipment but requires regulatory oversight and preventive strategies by employers and public health authorities to limit exposure to hazardous substances in the workplace (Mihalache et al., 2017; Schulte et al., 2018). Ultrafine particles can penetrate deep into the respiratory tract and deposit in the alveolar region, where they may accumulate, interact with the epithelium, or a small fraction can translocate into the bloodstream (Li et al., 2019; Kreyling et al., 2010).

However, evaluating the hazard potential of inhaled particles at the alveolar level remains challenging due to the limited physiological relevance of existing experimental exposure models. While animal models, particularly rodents, have been widely used for inhalation toxicity testing, anatomical and physiological differences from the human lung, e.g., absence of respiratory bronchioles and species-specific deposition patterns, limit their predictive value (Hofmann et al., 1989). Further, the lack of mechanistic insight into human-relevant toxicological insights has led to a shift in regulatory interest and validation efforts toward non-animal methods (Clippinger et al., 2018; Movia et al., 2020).

With recent advances in human cell culture techniques, *in vitro* models of the human alveolar epithelium have become increasingly accessible and sophisticated. These models have evolved from simple, submerged monolayers to engineered three-dimensional systems integrating defined mechanical, structural, and exposure cues, aiming to closely mimic native tissue while ensuring reproducibility and relevance for toxicological assessments (Artzy-Schnirman et al., 2021; Rothen-Rutishauser et al., 2008; Weber et al., 2026). Key improvements include the cultivation of alveolar epithelial cells at the air–liquid interface (ALI), which enables aerosol delivery *in vitro* and promotes cellular polarisation, surfactant production, and a more *in vivo*-like morphology, especially when using type II-like pulmonary epithelial cell lines, such as A549 cells (Barosova et al., 2021; Ohlinger et al., 2019; Wu et al., 2017). Similarly, bioinspired scaffolds, such as electrospun membranes, which structurally resemble the fibrous basement membrane, support epithelial barrier formation and physiological cell behaviour (Gonçalves et al., 2023). Further refinements include the application of physiological shear stress on the basal side to simulate blood perfusion, and mechanical stimulation in the form of cyclic stretch and/or controlled apical air flow to emulate breathing movements, which have been shown to o critically modulate epithelial permeability, barrier function and inflammatory signalling (Huh et al., 2010; Stucki et al., 2015; Nossa et al., 2019).

While the breathing motion can be easily simulated with microfluidic lung-on-chip devices, which are commercialised by different companies, they offer limited utility for modelling aerosol exposure to the alveolar epithelium (Huh et al., 2010; Artzy-Schnirman et al., 2019). Their size and closed or semi-enclosed architecture restricts direct access to the cultured tissue, fundamentally limiting compatibility with standard nebulisation devices and dosimetry approaches (Cei et al., 2021; Bannuscher et al., 2022; Frohlich et al., 2013). As a result, these systems may not reliably reproduce realistic aerosol exposure conditions, particularly at low doses relevant for occupational and environmental scenarios. Consequently, some chip-based studies still evaluate particle toxicity under submerged conditions, dispensing the test material as a liquid suspension onto the apical surface of the culture. This workaround compromises both deposited dose control and physiological relevance, particularly for low-dose aerosol exposures typical of occupational and environmental settings. Indeed, comparative studies consistently show that submerged exposure elicits a significantly weaker inflammatory or cytotoxic response compared to aerosol deposition at the ALI at comparable concentrations, underlining the necessity for aerosol-based, low-dose exposure approaches to achieve to achieve appropriate exposure sensitivity *in vitro* (Lenz et al., 2013; Loven et al., 2021). Similarly, micro- and nano-particle uptake, transepithelial transport, and inflammatory gene expression in lung cells have been reported to be amplified under cyclic stretch compared to static conditions, suggesting synergistic effects between mechanical strain and particulate stressors (Doryab et al., 2021; Schmitz et al., 2019). Collectively, the documented impacts of biomimetic scaffolds, cyclic stretching, perfusion, and aerosol exposure indicate that their controlled integration within a single experimental platform is essential for a comprehensive *in vitro* investigation of the adverse effects of inhaled particles.

Milli-fluidic bioreactors, like CIVIC and MALI, provide a complementary approach by preserving open and accessible culture geometries, as well as clinically relevant aerosolization surfaces which are likely less affected by border effect with respect to micro-sized devices (Mastrorocco et al., 2022; Cei et al., 2021; Doryab et al., 2021). In addition, commercially available exposure systems such as VITROCELL® and ALICE provide well-established solutions for aerosol generation, deposition, and dosimetry at the air–liquid interface. However, they are primarily designed as static exposure modules and do not incorporate basal fluidic perfusion or mechanical stimulation. Consequently, they fail to capture the dynamic mechanical and fluidic cues of the alveolar microenvironment, which are known to modulate epithelial responses (Leroux et al., 2022). Taken together, these limitations highlight that current systems involve inherent trade-offs between physiological mechanical actuation, realistic aerosol exposure, and quantitative dosimetry.

To address these limitations, we developed the Dynamic Air-Liquid Interface (DALI) system, an advanced milli-scale *in vitro* platform designed to replicate the mechanical complexity of the human alveolar environment, integrating: i) physiological cyclic strain of a biomimetic membrane, ii) direct aerosol delivery at the air–liquid interface, iii) continuous basal perfusion, v) real-time dosimetry.

Here we employed the system to investigate how dynamic physiological breathing conditions (5% stretch at a frequency of 0.2 Hz for 6 h) affect the response of human lung epithelial cells (A549) and demonstrate the feasibility and biological compatibility of the DALI platform. Then, using the same conditions, we examined transcriptional responses to aerosolised crystalline quartz silica (DQ_12_), a well characterized reference material in inhalation toxicology which is known to induce inflammation and fibrosis (Reuzel et al., 1991). A low DQ12 concentration was selected based on a guidance document for exposure-relevant dosing, which integrates material physicochemical properties with available *in vivo* and human exposure data.

## Materials and methods

### Materials

The A549-eGFP-Puro cell line was purchased from Imanis Life Sciences (Rochester, USA). All standard cell culture reagents were obtained from Gibco Life Technologies (Zug, Switzerland), unless otherwise specified. Cell culture flasks were purchased from TRP (Trasadingen, Switzerland). The 96-well plates (#3513, Corning, Reinach, Switzerland), 6-well plates (#353046), and cell culture inserts (#353181) were obtained from Falcon® (Vernier, Switzerland). Puromycin, Triton X-100, lipopolysaccharide (LPS), paraformaldehyde, bovine serum albumin (BSA), DAPI, Kaiser’s glycerol gelatin mounting medium, and sterile 0.9% sodium chloride solution were purchased from Sigma Aldrich (Buchs, Switzerland). The WST-1 reagent (#5015944001) was obtained from Roche Diagnostics (Rotkreuz, Switzerland), and crystalline quartz silica (DQ_12_) powder was acquired from the Institute of Reference Materials and Measurements (Geel, Belgium). The endotoxin quantification kit (#A39552), Rhodamine Phalloidin (#R415, Invitrogen™), AlexaFluor 647-conjugated goat anti-mouse antibody (#A21235, Invitrogen™), and Fast SYBR Green master mix (Applied Biosystems) were purchased via Thermo Fisher Scientific (Basel, Switzerland). Araldite® Rapid adhesive was purchased from Huntsman Advanced Materials (Basel, Switzerland). The anti-E-cadherin antibody (#sc-21791) was purchased from Santa Cruz Biotechnology (Heidelberg, Germany). The anti-paxillin antibody (#ab32084) and AlexaFluor 555-conjugated goat anti-rabbit antibody (#ab150078) were obtained from Abcam (Amsterdam, Netherlands). The anti-SP-B antibody (#840144) was acquired from R&D Systems (Minneapolis, USA). For RNA isolation and qPCR, the ReliaPrep™ RNA Cell Miniprep Kit and RNasin® Plus RNase inhibitor (#NZ2611) were purchased from Promega (Dübendorf, Switzerland), the Omniscript® RT Kit (#205113) from Qiagen (Hilden, Germany), and the oligo-dT primers from Microsynth (Balgach, Switzerland). Primers for qRT-PCR were purchased from Thermo Fisher Scientific (Zug, Switzerland) and are further specified in the Supplementary Material (Supplementary Table S1).

### Fabrication of Bionate® membranes

Bionate® II 80A, a commercial poly (carbonate)urethane copolymer, was selected for its excellent elasticity, oxidative stability, and compatibility with long-term culture conditions under dynamic strain. Membranes were fabricated using electrospinning, a technique that generates non-woven meshes with controlled porosity and fibre architecture. To prepare the polymeric spinning solution, Bionate® was dissolved at 10% w/v in 1,1,1,3,3,3-hexafluoro-2-propanol (HFP; Sigma-Aldrich) in a glass bottle under a fume hood overnight at room temperature on a magnetic stirrer to ensure complete dissolution. A 40 mL stock solution was typically prepared, yielding sufficient material for approximately ten membranes, with each membrane requiring 4 mL of solution. Electrospinning was performed using a Linari Engineering apparatus (Linari Eng, Italy). Before spinning, a sheet of aluminium foil was fixed to the grounded rotating collector using double-sided tape at three corners, with a fourth corner connected to the metal grounding contact. The collector-to-needle distance was set to 15 cm. The polymer solution was loaded into a 10 mL syringe fitted with a 0.41 mm inner diameter needle, mounted on the syringe pump, and connected to the high-voltage power supply via an alligator clip attached to the needle. Electrospinning parameters were set as follows: an applied voltage of 30 kV, a flow rate of 1 mL/h, and a total volume of 4 mL. Environmental controls included maintaining room temperature and standard atmospheric pressure, with active ventilation during operation. Upon completion, the fabricated membranes were left suspended in ambient conditions for 48 h to allow complete solvent evaporation. Circular membrane sections were traced using a membrane holder as a guide. Circles were typically drawn from the central region of the sheet to ensure selection of areas with uniform fibre density and optimal membrane thickness. Each electrospun sheet yielded approximately five usable membranes, depending on deposition homogeneity. Membrane discs were then carefully excised with sterile scissors and peeled from the aluminium substrate using forceps.

To improve cell adhesion, electrospun Bionate® membranes were coated with RTC. A coating solution was prepared by mixing 50% v/v RTC stock solution (3 mg/mL, ThermoFisher), 30.33% v/v DMEM supplemented with 10% v/v foetal bovine serum (FBS) and 1× penicillin/streptomycin (cell-specific culture medium for A549-GFP cells), and 19.66% v/v sterile-filtered 50 mM HEPES buffer (pH 7.0, ThermoFisher). All components were brought to room temperature and mixed thoroughly to achieve a final RTC concentration of 1.5 mg/mL. For each membrane (4.5 cm^2^), approximately 400 µL of coating solution were gently pipetted dropwise onto the surface to ensure uniform distribution. Due to the intrinsic hydrophobicity of the Bionate® substrate, membranes were gently tapped at the edge of the well plate to promote even coating across the entire surface. Coated membranes were incubated overnight at 37 °C to allow for collagen adsorption. If not used immediately, membranes were washed with PBS and stored at 4 °C under sterile conditions until further use.

### Characterisation of Bionate® membranes

The membrane’s morphological features–such as thickness, pore size and fibre diameter–were characterised using a FEI Quanta 450 FEG (Thermo Fisher Scientific) Scanning Electron Microscopy (SEM) and derived in ImageJ. Mechanical testing was conducted to assess the performance of the membranes under different conditions. Specifically, uniaxial tensile tests were conducted using a ProLine Z005 machine (Zwick Roell) equipped with a 10 N load cell, applying a constant strain rate of 0.1% s^-1^ until a 20% strain was achieved on 10 mm × 30 mm membrane samples. Young’s modulus was calculated from the linear region of the stress-strain curves in dry conditions, under wet incubation in PBS at 37 °C and after 7 days of incubation under cyclic stretching at 5% strain and 0.4 Hz.

Surface wettability was assessed by estimating the contact angle from images acquired using an optical tensiometer (Theta Lite - Biolin Scientific) with three fluids: deionised water (dH2O) as a standard reference, PBS 10X, and RPMI culture medium.

Membrane permeability was measured under hydrostatic pressure using Darcy’s Law. Briefly, the membrane is fixed on a bottleneck using a cap. The cap is cut in correspondence with its central region, allowing fluid flow through the membrane. The end of the bottle is cut, and a tube is fixed in correspondence with the bottleneck, ensuring diameter continuity from the cap to the tube. The tube is then filled with distilled water, maintaining a constant fluid level during the test.

Mechanical and structural test data are reported as mean ± standard deviation for at least three recorded values (n > 3). Statistical analysis was performed using the One-way ANOVA test, with a significance level of p < 0.05 for each test.

### Cell culture

For all experiments, the human lung carcinoma cell line A549-eGFP-Puro was used as an *in vitro* model of alveolar type II epithelial cells. This A549 variant is stably transduced with the lentiviral vector LV-eGFP-PGK-Puro (LV031), which drives expression of the enhanced green fluorescent protein (eGFP) under the control of the spleen focus-forming virus (SFFV) promoter and the puromycin resistance gene (Puro) under the phosphoglycerate kinase (PGK) promoter, resulting in constitutively fluorescent cells. Cells were maintained in Dulbecco’s Modified Eagle Medium (DMEM) supplemented with 10% heat-inactivated foetal bovine serum (FBS), 1% L-glutamine, 1% penicillin/streptomycin, and 1% puromycin. Cultures were incubated at 37 °C in a humidified atmosphere containing 5% CO_2_ and passaged every 3–4 days until they reached 80%–90% confluency.

### Cultivation of alveolar epithelial models

Physiological cultivation of *in vitro* lung models was achieved using the Dynamic Model for the ALveolar Interface (DALI) bioreactor in combination with electrospun, stretchable membranes fabricated from Bionate®, as previously described (PATROLS, 2025a). Membranes (4.5 cm^2^) were mounted into a custom-designed holder and sterilised in accordance with the Standard Operating Procedure for assembly and use of the DALI system (PATROLS, 2025b). The membrane holder, composed of PDMS-encapsulated neodymium magnets, was placed in a 6-well plate and immersed in a 70% ethanol solution in deionised water (v/v) for 15 min. It was then allowed to air dry under a laminar flow hood. The membrane was fixed between the magnetic rings and then subjected to the same ethanol immersion protocol, followed by two PBS washes to remove residual alcohol. Subsequently, membranes were exposed to ultraviolet (UV) light for 15 min per side to ensure complete surface decontamination. All other bioreactor components—including the bioreactor chambers, membrane, silicone tubing, and PDMS-sealed collector cap—were sterilised using one of three validated methods: autoclaving (excluding the magnets, which lose magnetisation above 80 °C), treatment with ethylene oxide, or surface disinfection using 70% ethanol followed by UV exposure. For the 3D-printed elements used to ensure air tightness, ethanol was avoided to prevent material degradation; these were exclusively sterilised by UV irradiation. Cells were seeded at a density of 1.1 × 10^6^ cells per membrane (corresponding to 2.44 × 10^5^ cells/cm^2^). The holder was placed in a standard 6-well plate, and the *in vitro* models were cultured for 5 days, with medium exchanged after 3 days in both the apical (1 mL) and basolateral (6 mL) compartments. Once full epithelial coverage of the membrane was confirmed following the corresponding guidance document, the apical medium was removed, and the holder was transferred into the DALI bioreactor (PATROLS, 2025c). *In vitro* models were maintained under air–liquid interface (ALI) conditions with continuous perfusion for 24 μL/min of medium in the basal compartment. Under these conditions, *in vitro* models are considered non-breathing (NB-condition). For breathing (B-) conditions, tissue cultures were exposed to an additional 6 h of cyclic stretch by applying pressurised air to the apical compartment, resulting in 5% stretch of the membrane at 0.2 Hz. Mechanical stimulation parameters were selected to reproduce physiological alveolar deformation during normal human breathing, in line with commonly adopted values in dynamic *in vitro* lung models. The stimulation was applied for 6 h to investigate acute cellular responses under breathing-like conditions while ensuring system stability.

### Preparation of DQ_12_ suspension

A stock suspension of DQ_12_ (2.56 mg/mL) was prepared for particle aerosolisation in accordance with the NANOGENOTOX dispersion protocol (Jensen and Dijkzeul, 2025). Briefly, 15.36 mg of DQ_12_ quartz was weighed using a precision balance (XA205 Dual Range, Mettler Toledo, Switzerland) inside a ventilated weighing cabinet and transferred to a sterile glass vial. Under sterile conditions, the powder was pre-wetted with 30 μL of absolute ethanol and then diluted to a final volume of 6 mL with endotoxin-free water. The resulting suspension was sonicated using a Branson Ultrasonics™ Sonifier™ SFX (Emerson, Dietzenbach, Germany) for either 22 min 3 s at 18% amplitude or 20 min 17 s at 17% amplitude, depending on equipment calibration. The stock was further diluted with endotoxin-free water to obtain working concentrations of 1 mg/mL and 250 μg/mL. Before exposure, DQ_12_ stock suspensions were confirmed to be endotoxin-free using the Pierce™ Chromogenic Endotoxin Quant Kit. The assay was performed according to the manufacturer’s protocol using the High Standard method, with a linear sensitivity range of 0.1–1.0 EU/mL.

### DQ_12_ dose response evaluation

Dose-response experiments were performed using the VITROCELL® Cloud 12 system (VITROCELL Systems GmbH, Waldkirch, Germany), which requires the use of standard 12-well cell culture inserts. Therefore, cell culture inserts were modified to replicate conditions comparable to those in the DALI system by incorporating Bionate® membranes. Inserts were sterilised with 70% (v/v) ethanol, and the original integrated membranes were removed using a scalpel. Circular sections of Bionate® membrane, matching the dimensions of the inserts (0.9 cm^2^), were cut and affixed using Araldite® Rapid adhesive. The modified inserts were sterilised, coated, and seeded with cells as described above. After 5 days of submerged cultivation, the inserts were transferred to the VITROCELL® system for aerosol exposure. Prior to exposure, the Cloud system was preheated to 37 °C with 2.5 mL of complete culture medium per well, and the quartz crystal microbalance (QCM) was connected to the oscillator. The Aeroneb® vibrating mesh nebuliser (MMAD 4.0–6.0 μm, Aerogen, Galway, Ireland) was prepared by sonication in 70% (v/v) ethanol for 15 min (37 kHz, power 50), followed by three rinses with 200 μL of 1% (v/v) isotonic NaCl in endotoxin-free water. DQ_12_ exposure was conducted under sterile conditions as previously described (Bannuscher et al., 2022; Braakhuis et al., 2023). In brief, the nebulisation protocol (Aerogen® Pro-X controller) included a 1 min pre-exposure period to stabilise the QCM signal, a 6 min nebulisation phase, 1 min of lid removal to allow QCM drying, followed by lid replacement, and an additional 3 min before data acquisition was stopped. For each exposure experiment, 70 μL of different DQ_12_ suspensions were aerosolised. DQ_12_ suspensions of 0.25 mg/mL, 1 mg/mL, 2.56 mg/mL and 2 × 2.56 mg/mL were tested. For negative controls, cells were exposed to 70 μL of nebulised 1% (v/v) isotonic NaCl in endotoxin-free water. The nebuliser was cleaned after each run by rinsing it three times with 200 μL of 1% (v/v) isotonic NaCl solution. After exposure, inserts were transferred back to a 12-well plate and maintained under air–liquid interface (ALI) conditions for an additional 24 h. As a positive control for the WST-1 assay, cells were treated with 0.2% Triton-X 100 under submerged conditions. The deposited mass of DQ_12_ was calculated by subtracting the QCM signal value at the end of the exposure from the value at the beginning of the stable signal phase, correcting for the signal attributable to 1% NaCl alone.

### DQ_12_ exposure in the DALI bioreactor

DQ_12_ exposure in the DALI system was adapted from the protocol established for the VITROCELL® Cloud 12 system, employing the same Aeroneb® vibrating mesh nebuliser. To maintain physiological temperature, the DALI bioreactor containing the cultured membrane was placed on a heating plate (IKA® RTC basic, Thermo Fisher Scientific, Basel, Switzerland) set to 37 °C. The nebuliser was mounted in an airtight configuration onto the bioreactor, and 10 μL of the 2.56 mg/mL DQ_12_ stock suspension was aerosolised, resulting in a deposition of approx. 0.31 μg/cm^2^ DQ_12_.

The exposure dose of 0.31 μg/cm^2^ is in alignment with occupationally extrapolated *in vitro* dose ranges established in the field for respirable crystalline silica: The framework for deriving occupationally relevant *in vitro* doses for inhaled particles was formalised by Gangwal et al. (2011), who used the Multiple Path Particle Dosimetry (MPPD) model to calculate alveolar mass retained per unit alveolar surface area following simulated occupational inhalation at regulatory exposure limits, and used these values to define the upper and lower bounds of biologically meaningful *in vitro* test concentrations. Applying this framework to respirable crystalline silica at the Occupational Safety and Health Administration (OSHA)/National Institute for Occupational Safety and Health (NIOSH) permissible exposure limit of 50 μg/m^3^ as an 8-h time weighted average (TWA) (NIOSH, 2019) and using a light exercise breathing pattern consistent with International Commission on Radiological Protection (ICRP) reference worker parameters, MPPD modelling yields an alveolar surface dose of 0.1–1 μg/cm^2^ for acute single-shift exposures. This range has been explicitly adopted as the occupationally relevant *in vitro* dose window for DQ12 in the PATROLS EU framework guidance for engineered nanomaterials lung dosing, which specifies an equivalent *in vitro* dose range of 0–1 μg/cm^2^ for DQ12 at the ALI based on *in vivo* extrapolation (PATROLS, 2025d).

Vehicle controls were exposed to 1% (v/v) isotonic NaCl in endotoxin-free water under identical conditions. As a positive control for the WST-1 assay, cells were treated with 0.2% Triton X-100 under submerged conditions. *In vitro* models were cultured for 24 h after exposure under dynamic (medium flow) conditions and 6 h of stretching (5%, 0.2 Hz) for B-conditions before subsequent analyses.

### Confocal laser scanning microscopy

For all fluorescence microscopy analyses, *in vitro* models cultured on Bionate® membranes were carefully removed from the holder, rinsed twice with PBS, and fixed with 4% (w/v) paraformaldehyde in PBS for 30 min at room temperature. Following fixation, samples were washed three times with PBS and permeabilised using 0.2% (v/v) Triton X-100 in 1% (w/v) bovine serum albumin (BSA) in PBS for 15 min, followed by three additional PBS washes. For cytoskeletal and nuclear staining, cells were incubated for 1 h with Rhodamine Phalloidin (1:100) and DAPI (1:50) diluted in PBS. For immunostaining, the Triton X-100/BSA solution was applied for 30 min to block nonspecific binding sites. Samples were then incubated for 2 h with primary antibodies diluted 1:200 in blocking buffer: rabbit anti-paxillin, mouse anti-E-cadherin, or mouse anti-surfactant protein B (SP-B). After three PBS washing steps, appropriate secondary antibodies were applied for 1 h: goat anti-rabbit Alexa Fluor 555-conjugated secondary antibody for paxillin, and goat anti-mouse Alexa Fluor 647-conjugated secondary antibody for E-cadherin and SP-B. Following a final wash, samples were mounted in Kaiser’s glycerol gelatin mounting medium. Imaging was performed using an inverted laser scanning confocal microscope (LSM, Stellaris 5, Leica, Germany) equipped with Power HyD S detectors and a Plan-Apochromat 63x/1.4 Oil CS2 objective (Leica, Switzerland), operated *via* LAS X software (Leica). The following laser excitation wavelengths were used: 405 nm (DAPI), 561 nm (Rhodamine Phalloidin and Alexa Fluor 555), 488 nm (eGFP), and 633 nm (Alexa Fluor 647).

### Image analysis

Images were processed using the Fiji distribution of ImageJ (version 1.53t) (Schmidt et al., 2018) to quantify cell orientation angle relative to the vertical axis, aspect ratio (major/minor axis) and epithelial layer thickness. Segmentation of cellular contours was performed semi-automatically, and morphological measurements were extracted for each condition. Image processing for nuclei-based cell counting was conducted using the Fiji distribution of ImageJ (version 1.53t) (Schmidt et al., 2018). DAPI-channel images (field size: 184.7 × 184.7 μm) were extracted and analysed using the StarDist plugin for automated nuclear detection and segmentation. A size threshold of >400 μm^2^ was applied to exclude cell debris and non-cellular artefacts. Cell counts were determined from three biological replicates per condition (one image analysed per replicate and condition) and extrapolated to the full membrane area (4.5 cm^2^) by multiplying the counted values by a scaling factor of 13,191.03.

### Cell viability assay

The WST-1 assay was used to spectrophotometrically quantify cellular metabolic activity as an indicator for cell viability. Following the removal of the culture medium and a PBS wash, the *in vitro* models were incubated for 45 min with the WST-1 reagent diluted 1:10 in complete medium. A volume of 750 μL of the WST-1 solution was applied to each membrane. Subsequently, 100 μL of the resulting supernatant was transferred in triplicate into a 96-well plate for absorbance measurement at 440 nm using either a Benchmark microplate reader (Bio-Rad, Cressier, Switzerland) or a Synergy H1 plate reader (BioTek, Agilent Technologies, Basel, Switzerland). The blank consisted of WST-1 reagent in medium without cells, and the positive control consisted of *in vitro* models treated with 0.2% Triton X-100 under submerged conditions. Metabolic activity is presented as a percentage relative to the negative control. For statistical analysis, data were normalised to the vehicle-exposed control (dose-response experiment) or non-breathing condition (in experiments in the DALI bioreactor).

### Reverse transcription-quantitative polymerase chain reaction

For RNA isolation, *in vitro* models were carefully removed from the holder and transferred to a 6-well plate, followed by washing with ice-cold PBS. Total RNA was extracted using the ReliaPrep™ RNA Cell Miniprep Kit according to the manufacturer’s protocol. RNA yield and purity were assessed using a NanoDrop™ 2000 spectrophotometer and associated software (Thermo Fisher Scientific, Zug, Switzerland). Isolated RNA was stored at −80 °C until further use. Complementary DNA (cDNA) synthesis was performed using the Omniscript® RT Kit, oligo-dT primers, and RNasin® Plus RNase inhibitor. RNA was diluted to 30.77 ng/μL in nuclease-free water, and 6.5 μL (200 ng) were used per reaction. Reverse transcription was carried out at 37 °C for 60 min in a thermal cycler, and the resulting cDNA was stored at −20 °C. Quantitative real-time PCR (RT-qPCR) was performed using 2 μL of two-fold diluted cDNA, 5 μL of SYBR Green Master Mix, and 4 μL of gene-specific primer mix. No-template controls were included for each gene to detect potential contamination or non-specific amplification. The housekeeping gene YWHAZ was used as an internal reference and included in each run. PCR was carried out on an Applied Biosystems 7,500 system with Sequence Detection Software v2.3 (Thermo Fisher Scientific, Waltham, MA, USA) using the following thermal cycling conditions: initial holding at 95 °C for 20 s, followed by 40 cycles of 95 °C for 10 s and 60 °C for 30 s, and a melt curve analysis (95 °C for 15 s, 60 °C for 1 min, 95 °C for 15 s, and 60 °C for 15 s). All samples were run in triplicate.

### Statistical evaluation

All results are based on three independent biological replicates unless stated otherwise. Data were analysed using GraphPad Prism (version 9.0.2, GraphPad Software, La Jolla, CA, USA) and are reported as mean values ±standard deviation (SD). Statistical significance was assessed using one-way analysis of variance (ANOVA), followed by Dunnett’s or Tukey’s *post hoc* test, as appropriate. For RT-qPCR data, statistical analysis was conducted using ΔCt values. The normality of the data was first assessed using the Shapiro–Wilk test. If the assumption of normal distribution was met, one-way ANOVA with Dunnett’s *post hoc* comparison was performed. A p-value below 0.05 was considered statistically significant (p values: * = 0.05; ** = 0.01).

## Results

### Characterisation of biomimetic membranes

To the best of our knowledge, DALI is currently the only milli-scale *in vitro* lung platform that combines breathing-like mechanical actuation, basal perfusion, biomimetic membranes, ALI aerosol exposure and real-time dosimetry within a single experimental framework, enabling controlled investigation of particle–epithelium interactions under dynamic conditions; other systems only partially combine these features (Huh et al., 2010; Stucki et al., 2015; Nossa et al., 2019; Artzy-Schnirman et al., 2019). The design and engineering of the DALI bioreactor is described in the PATROLS Annex 3,202 and summarised in the Supplementary Material (Supplementary Figure S1).

**FIGURE 1:**
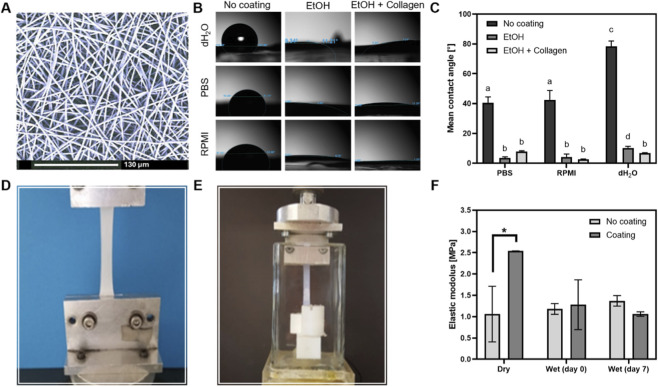
Characterisation of electrospun Bionate® membranes. **(A)** SEM micrograph of fibres. **(B)** Images of droplets on membranes with different surface treatments using different fluids ((H_2_O), PBS, cell culture medium). **(C)** Contact angle data. **(D)** Samples with grips during dry tensile mechanical tests set up in dry conditions. **(E)** Set-up with liquid chamber for mechanical testing in wet conditions. **(F)** Elastic moduli for dry and wet conditions, with and without collagen coating. Different letters and * indicate statistical significance (p < 0.05).

To simulate the physiological niche of the lung extracellular matrix, a cell culture membrane was fabricated *via* electrospinning using the Bionate® polymer, and analysed *via* scanning electron microscopy (SEM) (Figure 1A), showing a homogeneous, randomly oriented fibrous structure. The average fibre diameter and pore diameter measured 2.4 ± 0.6 µm and 4.3 ± 1.9 µm, respectively, and total membrane thickness was 54.1 ± 10.7 µm. Water, phosphate-buffered saline (PBS) and cell culture media contact angles were measured to assess wettability of the membranes with different coatings (Figures 1B,C). While membranes without coating exhibited high contact angles, they were significantly reduced in coated samples, irrespective of the fluid used. Permeability tests indicated high diffusion capabilities of the membrane with K = 2.71 ± 0.74 × 10^−13^ m^2^. Importantly, the surface treatments used to increase wettability and collagen coating enhanced cell–material interactions and did not occlude the porous network or compromise membrane permeability. Mechanical characterisation of the membranes was conducted under three experimental conditions: dry, wet (PBS at 37 °C), and wet post-incubation (7 days, at 37 °C) (Figures 1D–F). In dry conditions, the membranes exhibited an elastic modulus of approximately 1.10 MPa. Coated samples showed a slight increase in stiffness under dry conditions, likely due to the densification of the collagen matrix. However, under hydrated and dynamic conditions, mechanical properties were not altered with respect to the dry condition without coating, and no evidence of plastic deformation or material degradation was observed.

### Morphological adaptation of A549 to cyclic stretching

A549 cells were seeded on Bionate® membranes and cultured for 5 days under submerged conditions to ensure epithelial monolayer formation and complete coverage of the membrane surface. The seeded membranes were then transferred to the bioreactor and cultured for 24 h under ALI conditions, with continuous medium flow in the basal compartment. For breathing (referred to as “B”) conditions, the cells were subsequently exposed to pressurised air in the apical compartment for 6 h, resulting in a cyclic multiaxial stretching of the tissue (5% stretch, 0.2 Hz). As a control, non-breathing (referred to as “NB”) models were cultured in the bioreactor for the same time without stretching. Both B- and NB-conditions included continuous perfusion of cell culture medium in the basal compartment.

**FIGURE 2:**
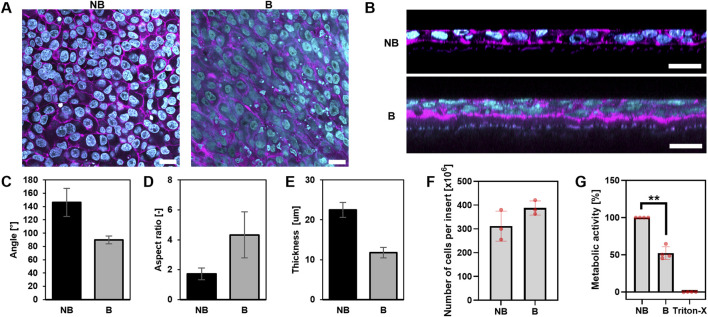
**(A)** A549 lung cell tissue morphology. Confocal laser scanning microscopy of cells cultured on membranes under NB and B (5% stretch, 0.2 Hz for 6 h) conditions. Cells were stained with DAPI (blue) and Phalloidin (pink) to visualise cell nuclei and f-actin, respectively. Scale bars 20 µm. **(B)** Cross sections of fluorescence microscopy of tissue cultures (DAPI: blue, Phalloidin: pink). Scale bars 20 µm. Quantitative analyses of **(C)** cell orientation angle, **(D)** aspect ratio, and **(E)** epithelial layer thickness. **(F)** Number of cells per insert. The cell number was determined based on micrographs of DAPI-stained nuclei and extrapolated to the growth area of 4.5 cm^2^. **(G)** Cell viability measured using the WST-1 assay.

Cell morphology was assessed using confocal laser scanning microscopy, visualising nuclei and the F-actin cytoskeleton via DAPI and Phalloidin staining, respectively (Figure 2A). Orthogonal micrographs show the zx and zy cross-sections of tissues cultured under NB- and B-conditions (Figure 2B). Under NB-conditions, phalloidin staining reveals F-actin filaments predominantly localised at the cell periphery. In contrast, under B-conditions, F-actin staining is more intense towards the basal side of the cell layer. A notable change in cell morphology occurs under B-conditions, where cells exhibit elongation and directional alignment of cell bodies. Morphometric analysis revealed that A549 cells aligned along the direction of mechanical strain, with mean orientation angles approaching 90°, significantly higher compared to NB-conditions (Figure 2C). These cells also displayed a significantly increased aspect ratio (Figure 2D). In line with cell alignment and elongation, the epithelial layer thickness was significantly reduced under B-conditions (Figure 2D). The number of cells per tissue culture was estimated from fluorescence microscopy images by counting DAPI-stained nuclei per field of view and extrapolating the number to the total membrane area (4.5 cm^2^) (Figure 2E). Under NB-conditions, approximately 4.1 × 10^6^ cells were detected per membrane, while tissues under B-conditions contained approximately 5.1 × 10^6^ cells. Although this represents a ∼25% increase in cell number under B-conditions, the difference was not statistically significant due to the relatively high variability observed among the NB samples. Metabolic activity was significantly reduced under B-conditions compared to the NB-condition (Figure 2F).

### Expression of cellular cohesion and focal adhesion associated proteins

**FIGURE 3:**
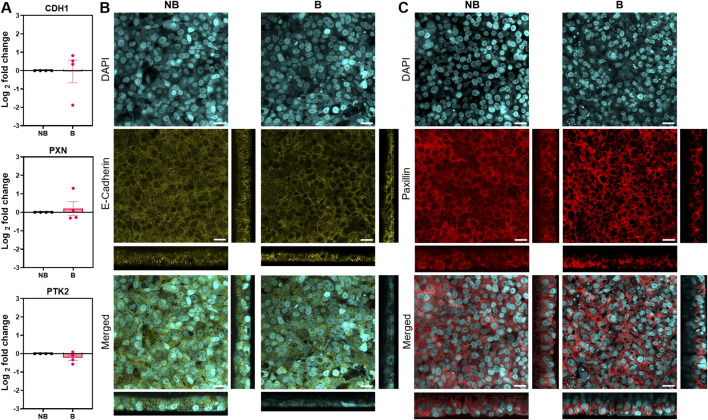
Effect of static cultivation and cyclic stretching on epithelial junctions and focal adhesions. **(A)** mRNA expression of E-cadherin (CDH1), paxillin (PXN), and focal adhesion kinase (PTK2) in A549 epithelial models after 6 h of NB- or B-conditions (5% cyclic stretch, 0.2 Hz), assessed by qRT–PCR. Data are normalised to YWHAZ and presented as mean ± SD (n = 4 biological replicates) **(B)** Confocal micrographs showing nuclei (DAPI, blue) and immunostaining of E-cadherin (yellow) or **(C)** paxillin (red) in A549 models under NB and B- conditions. Scale bars, 20 μm. Statistics: Data are normalised to YWHAZ.

A549 cells grown under NB- and B-conditions were further evaluated regarding the expression of proteins relevant to cell-cell interactions and focal adhesions at both the gene and protein levels (Figure 3). The expression of the *CDH1* gene was overall not significantly altered under B-conditions when compared to the NB-group (Figure 3A). Micrographs of immunofluorescence staining also do not show quantitative differences in E-cadherin expression between the two groups (Figure 3B). However, under B-conditions, the protein appears to exhibit stronger localisation along the borders between neighbouring cells. The expression of the focal adhesion protein paxillin *(PXN)* and the associated *focal adhesion tyrosine kinase (PTK2)* was assessed in NB- and B-conditions (Figure 3A). No significant change was observed on mRNA level for *PXN* or *PTK2*. Fluorescence staining of paxillin (Figure 3C) showed that the protein was shifted from a diffuse cytoplasmic distribution to discrete basal foci under B-conditions.

### Characterisation of secretory function and inflammatory response

**FIGURE 4:**
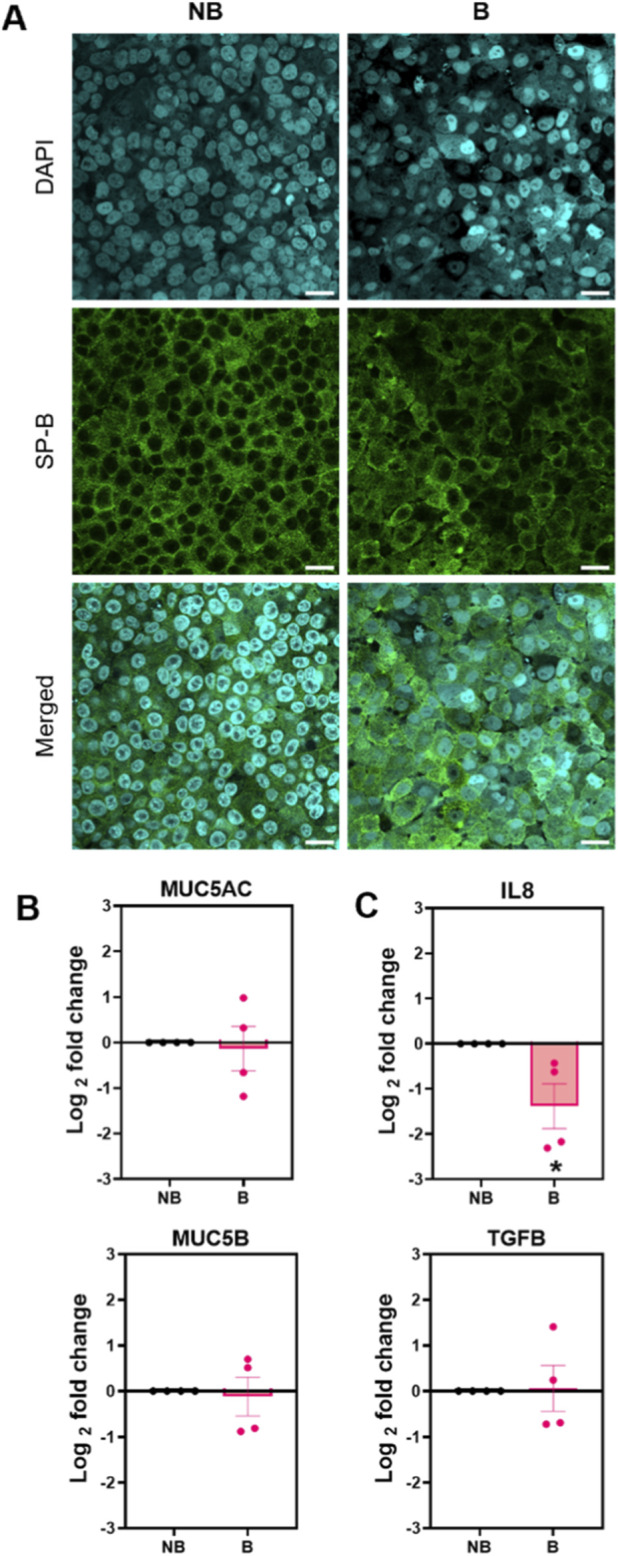
Evaluation of A549 tissue models with respect to surfactant protein expression, mucus production, cytokines, and stress signalling. **(A)** Confocal laser scanning microscopy images of A549 epithelial models cultured under NB and B- conditions (5% cyclic stretch, 0.2 Hz, 6 h), showing nuclei (DAPI, blue) and immunostaining for surfactant protein B (SP-B, green). Scale bars: 20 µm. **(B)** mRNA expression of mucin 5AC (MUC5AC) and mucin 5B (MUC5B) under NB and B- conditions, assessed by qRT-PCR. Data are normalised to YWHAZ and presented as mean ± SD (n = 4 biological replicates. **(C)** mRNA expression of interleukin-8 (IL8) and transforming growth factor beta (TGFB) under NB and B-conditions, assessed by qRT-PCR. Data are normalised to YWHAZ and presented as mean ± SD (n = 4 biological replicates).

The effect of cyclic stretch on the cells was further assessed with respect to surfactant protein B (SP-B), mucin and epidermal growth-factor receptor (*EGFR*) expression, as well as stress response (Figure 4). Immunofluorescence staining (Figure 4A) revealed that under NB- conditions, A549 cells exhibited a homogeneous cytoplasmic distribution of SP-B, with small vesicular structures visible within the cytosol. In contrast, under B-conditions, SP-B staining appeared weaker overall. Expression of the mucin genes *MUC5AC* and *MUC5B* was assessed *via* qRT-PCR (Figure 4B) and was slightly reduced under B- compared to NB-conditions. *EGFR* expression showed a modest increase under B-conditions (Supplementary Figure S3A). qRT-PCR was performed to analyse the expression of representative genes involved in inflammation (*IL8*), fibrosis and tissue remodelling (*TGFB*) (Figure 4C), as well as oxidative stress (*HMOX1*) (Supplementary Figure S3A). *IL8* expression was significantly decreased in B-conditions compared to NB-conditions. Neither *TGFB* nor *HMOX1* expression showed substantial variability in B-conditions.

### System integration with real-time QCM

**FIGURE 5:**
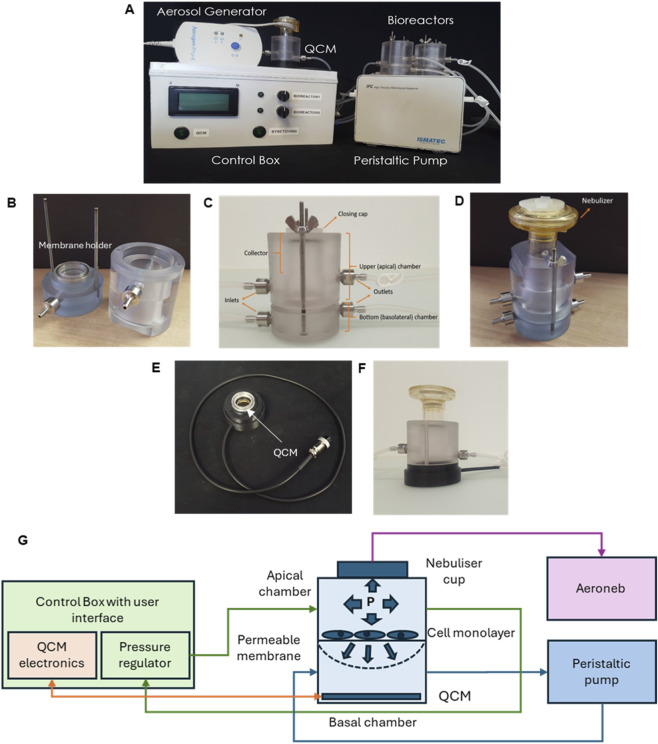
DALI system, integrating bioreactors, peristaltic pump, aerosol generator QCM and a control box. **(A)** control hardware and pump. Details of the bioreactor, highlighting **(B)** membrane holder separating the apical and basal chambers; **(C)** tube inlets/outlets for the two chambers; **(D)** coupling with the Aeroneb nebulizer**,** on top on the apical chamber. Details of the QCM module, outlining **(E)** the basal module and the QCM crystal, **(F)** bioreactor apical chamber attached to the QCM unit, **(G)** schematic representation of module integration, membrane deformation and cell monolayer on the apical side of the membrane.

The DALI system demonstrated reliable and reproducible operation across all integrated modules, with no evidence of cross-contamination between parallel chambers during long-term dynamic culture. Media flow was ensured *via* peristaltic pumping system (Figures 5A,B), while the control unit allowed precise adjustment of membrane strain *via* electropneumatic regulators. This allowed replicating physiological breathing cycles (at 0.2 Hz) with linear strain levels ranging from 5% to 17%. Real-time monitoring of pressure and mechanical deformation through an integrated LCD interface (Figure 5A) confirmed the accuracy of actuation and stable long-term operation. The aerosol exposure system performed effectively through direct coupling with a commercial Aeroneb Pro nebulizer (Figure 5C). During experimental validation, the system maintained both air and liquid tightness over time. The QCM’s geometry matched that of the DALI bioreactor (Figure 5D), ensuring comparable deposition profiles. Further details on system integration and operation are report in Supplementary Figures S1, 2.

The DALI system (Figures 5A–G) demonstrated reliable and reproducible operation across all integrated modules, with no evidence of cross-contamination between parallel chambers during long-term dynamic culture. Media flow was through a peristaltic pump, while the control unit allowed precise adjustment of membrane strain *via* electropneumatic regulators (Figure 5A). This allowed replicating physiological breathing cycles (at 0.2 Hz) with linear strain levels ranging from 5% to 17% in the bioreactor (Figures 5B–D). Real-time monitoring of pressure and mechanical deformation through an integrated LCD interface (Figure 5A) confirmed the accuracy of actuation and stable long-term operation. The aerosol exposure system performed effectively through direct coupling with a commercial Aeroneb Pro nebulizer (Figure 5D). During experimental validation, the system maintained both air and liquid tightness over time. The QCM’s geometry matched that of the DALI bioreactor (Figures 5E,F), ensuring comparable deposition profiles. Figure 5G schematizes the integration of the different DALI modules, highlighting the direction of membrane deformation due to overpressure in the apical chamber. Further details on system integration and operation are report in the Supplementary Material.

### Exposure to low-dose aerosolised silica

**FIGURE 6:**
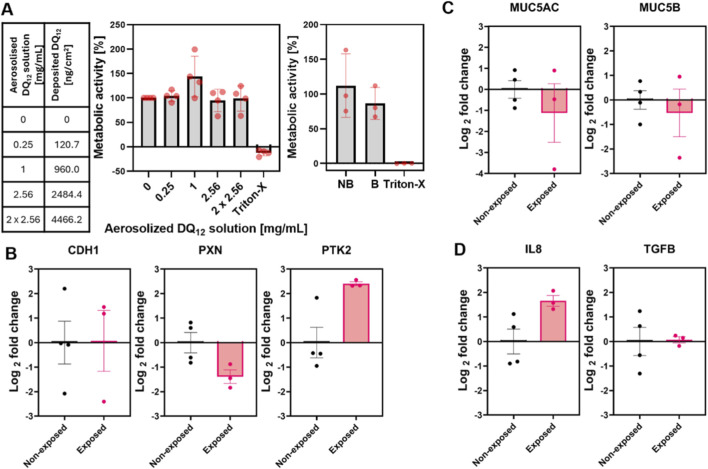
DQ12 exposure and cellular response. **(A)** A549 cultures exposed to aerosolised DQ12 to investigate dose-response under NB-conditions and effect of chosen concentration under B-conditions (10 μL of 2.56 mg/mL solution, resulting in 0.31 μg/cm^2^ deposited DQ12, 5% cyclic stretch, 0.2 Hz, 6 h), assessed by WST-1 assay. **(B)** mRNA expression of cell junction and focal adhesion markers: E-cadherin (CDH1), focal adhesion kinase (PTK2), and paxillin (PXN) for exposed and non-exposed tissues under B-conditions. **(C)** mRNA expression of mucin-associated genes and regulators: mucin 5AC (MUC5AC), mucin 5B (MUC5B), and epidermal growth factor receptor (EGFR) for exposed and non-exposed tissues under B-conditions. **(D)** mRNA expression of stress response markers: interleukin-8 (IL8), transforming growth factor beta (TGFB), and heme oxygenase 1 (HMOX1) for exposed and non-exposed tissues under B-conditions. Statistics: Statistical significance was evaluated using ANOVA followed by Dunnett’s or Tukey’s *post hoc* test, as appropriate. For qRT–PCR, data are normalised to YWHAZ and shown as mean fold changes with respect to non-exposed cells under B-conditions ±SD (n = 4 for non-exposed samples, n = 3 for exposed samples, except EGFR and HMOX1, where n = 2).

Before investigating DQ_12_ exposure in the DALI bioreactor, a dose–response study was performed on A549 cells under static conditions (ALI culture on collagen coated Bionate® membranes, no cyclic stretching) to evaluate the effect of different aerosolised DQ_12_ concentrations. Cell viability of the cultures after DQ_12_ exposure was compared to a vehicle control (1% NaCl; 0 ng/cm^2^ DQ_12_) by using the WST-1 assay to measure metabolic activity (Figure 6A). Suspensions with 70 μL of 0.25 mg/mL, 1 mg/mL, 2.56 mg/mL and 2 × 2.56 mg/mL were aerosolised in the VITROCELL® Cloud 12 system, resulting in deposition of 0.12, 0.96, 2.48, and 4.47 μg/cm^2^. These concentrations were chosen to bracket a physiologically realistic range of concentrations while remaining well below the cytotoxic thresholds reported for studies assessing DQ_12_ toxicity under submerged conditions (Cakmak et al., 2004). After normalisation to the vehicle control, none of the tested concentrations of DQ_12_ measurably impaired cell viability. Based on these results, exposure experiments were subsequently conducted within the DALI system under NB- and B-conditions by aerosolising a single dose of 10 μL with 2.56 mg/mL DQ_12_ suspension, corresponding to approximately 0.31 μg/cm^2^ deposited DQ_12_. The A549 tissue model was analysed 24 h after exposure to aerosolised DQ_12_. Metabolic activity from three biologically independent replicates showed substantial variability, but there was no significant decrease in viability between the NB- and B- conditions. We thus focused on the effects of DQ_12_ exposure on cells in B-conditions by analysing gene expression related to cell junctions and focal adhesions, mucin secretion, as well as stress response (Figures 6B–D), comparing the data with the non-exposed B-condition. A comparative analysis of mRNA expression between NB- and B conditions after DQ_12_ exposure is presented in the Supplementary Material (Supplementary Figure S4).

DQ_12_ exposure had no measurable effect on *CDH1* expression compared to the non-exposed control (Figure 6B). *PXN* expression slightly decreased, while *PTK2* exhibited upregulation, although neither change was statistically significant. The effects of DQ_12_ exposure on the expression of mucus genes *MUC5AC* and *MUC5B* (Figure 6C) and *EGFR* (Supplementary Figure S3B) were not statistically significant. Regarding inflammatory response, *TGFB* remained unchanged, whereas *IL8* was upregulated after DQ_12_ exposure (Figure 6D). *HMOX1* expression was notably downregulated in cells exposed to DQ_12_, but due to the limited number of biological replicates for this gene, statistical significance could not be confirmed (Supplementary Figure S3B).

## Discussion

The DALI bioreactor constitutes a modular and physiologically relevant platform for investigating the biological effects of aerosolised particles in lung cells *in vitro* under controlled, breathing-like mechanical conditions. Designed to emulate key aspects of the alveolar microenvironment, DALI addresses a technological gap by coupling within a single *in vitro* device:

- physiological cyclic strain thanks to apical chamber pressurisation;
- biomimetic, porous, sterchable and biocombatible membrane fabricated *via* bionate electrospinning;
- direct aerosol delivery at the air–liquid interface, thanks to the press fit integration of a nebuliser cap on the apical chamber of the bioreactor and its connection with a commercial aerosol generator;
- continuous basal perfusion, through connection with a peristaltic pump;
- real-time dosimetry, with a QCM sensor integrated in the basal chamber and connected with the electronics in the control box.

This integration enables systematic interrogation of particle–cell interactions under exposure scenarios that are more physiologically relevant than static or submerged systems, positioning DALI as a versatile tool for inhalation research.

The biomimetic electrospun Bionate® membranes provided a homogeneous fibrous morphology that closely resembles the porous and disordered architecture of the native lung extracellular matrix (ECM) (Suki and Bates, 2011). The average fibre and pore diameters lie within the range reported for lung ECM fibres (100 nm–10 µm) and pores (2–10 µm), while the membrane thickness, although greater than that of the alveolar basement membrane (≈0.1–0.5 µm), is consistent with values commonly used in lung *in vitro* models to ensure mechanical integrity during cyclic actuation (Stucki et al., 2015).

In addition to microstructural similarity, membrane wettability and permeability are key parameters for epithelial culture. Ethanol treatment followed by rat-tail collagen coating substantially reduced the contact angle and thereby improved the effective wettability of the surface. This is relevant not only for water, but also under physiologically more meaningful conditions, since PBS reflects ionic interactions at the interface and cell culture medium captures the protein-mediated wetting behaviour experienced by cells (Vogler, 2012). Importantly, these surface treatments enhanced cell–material interactions without occluding the porous network or compromising membrane permeability, and mechanical testing confirmed that biological functionalisation did not adversely affect membrane stability under dry or hydrated conditions (Nichols et al., 2014; Stucki et al., 2015). Altogether, this balance of permeability, bioactivity, and mechanical robustness supports the suitability of the membrane for simulating physiological breathing dynamics *in vitro*.

DALI’s characteristics with respect to other state of art *in vitro* lung models are summarized in Table 1. The table highlights that DALI is currently the only system which integrates a biomimetic membrane with flow, physiological stretching, aerosol delivery and real-time dosimetry.

**TABLE 1:** Characteristics of DALI compared with other advanced *in vitro* lung models.

Design feature	DALI	Other advanced system
Membrane	Elastic electrospun fibrous membrane (Bionate®), ECM-like, stretchable and permeable	Rigid porous membranes (e.g., PET) or thin elastomeric films with limited biomimicry
Culture area	Large milliscale area (∼4.5 cm^2^), clinically relevant and easily accessible	Microscale culture area (mm^2^ range), limited surface and difficult access in lung-on-chip systems. Similar culture area in other millifluidic bioreactors
Strain capability	Physiological cyclic out of plane multiaxial strain (5%–17%, 0.2 Hz), pressure-driven	In plane and out of plane multiaxial strain (5%–15%, 0.1–0.3 Hz), pressure or vacuum-driven
Perfusion	Continuous basal perfusion (0.05–2 mL/min)*	0.0003–0.005 mL/min in microfluidifc systems, comparable to DALI in other millifluidic devices
Dosimetry integration	Integrated real-time QCM for dose monitoring	Integrated dosimetry or indirect dose estimation absent
Aerosol compatibility	Direct compatibility with clinical nebulizers and aerosol exposure at ALI.	Limited aerosol compatibility or indirect/submerged particle delivery
Integration	Yes	Partial**

* While blood flow in individual pulmonary capillaries is in the order of a few microliters per minute, *in vitro* platforms represent multiple capillary equivalents over a defined tissue area. Consequently, higher basal flow rates are required to ensure adequate nutrient turnover.

** While some *in vitro* lung models integrate perfusion with mechanical stretching and/or aerosol delivery, quantitative and real-time dosimetry is rarely implemented under dynamic conditions.

Using this platform, we show that the introduction of low-amplitude cyclic strain (5%, 0.2 Hz) is sufficient to modulate epithelial organisation and function on elastic fibrous scaffolds, inducing cytoskeletal reorganisation and metabolic adaptation without eliciting overt stress responses. Under breathing conditions, A549 cells exhibited elongation and directional alignment, with significantly increased orientation angles and aspect ratios compared with non-breathing controls. These morphological changes are consistent with a mechanoresponsive phenotype and with previous reports showing alignment and elongation of A549 cells in response to cyclic deformation (Roshanzadeh et al., 2020; Ohashi et al., 2017; Nossa et al., 2021). The reduction in epithelial layer thickness observed under breathing conditions further suggests tissue compaction and structural reorganisation in response to mechanical cues, consistent with reports that cyclic strain promotes cytoskeletal alignment, junctional maturation, and enhanced epithelial organisation in lung models (Stucki et al., 2015; Huh et al., 2010).

The shift in F-actin organisation and the redistribution of paxillin toward discrete basal foci under breathing conditions support the interpretation that cyclic strain induced active remodelling of the cell–matrix interface. Paxillin is known to respond to externally applied mechanical stress primarily through redistribution within focal adhesions rather than through changes in gene expression (Sero et al., 2011). Indeed, while PXN and PTK2 transcript levels were not significantly altered, paxillin staining changed from a diffuse cytoplasmic pattern to a more focal basal localisation, suggesting enrichment at adhesion sites. This is in line with studies showing that lower stretching forces promote paxillin clustering at the cell periphery and focal adhesions, whereas stronger mechanical loads can trigger more pronounced redistribution toward perinuclear regions (Thomas et al., 2006; Gawlak et al., 2014). Similarly, although CDH1 expression remained unchanged, the more pronounced localisation of E-cadherin at cell borders under breathing conditions may indicate enhanced cohesion between neighbouring cells in response to cyclic actuation (Verma et al., 2015; Jaworski et al., 2025). Together, these findings suggest that the applied mechanical stimulus primarily affected epithelial organisation at the structural and subcellular level rather than through major early transcriptional shifts in adhesion-related genes.

The approximately 25% higher cell number estimated for breathing conditions likely reflects this altered cellular organisation rather than true proliferation during the 6 h stimulation period. The more elongated morphology of cells under cyclic stretch may allow denser packing across the scaffold, whereas the relatively short duration of stimulation and the concomitant reduction in metabolic activity argue against a substantial proliferative contribution. Although studies directly comparing viability or metabolic activity between static and cyclically stretched A549 cultures remain limited, previous work has generally reported no detrimental effects of physiological stretch on cell viability, while nevertheless observing enhanced cytokine release or superoxide production under stronger or more prolonged stimulation (Radiom et al., 2020; Vlahakis et al., 1999; Chapman et al., 2005). In primary epithelial cells, moderate and high strain amplitudes have been shown to reduce viability in a strain-dependent manner (Hammerschmidt et al., 2004). In the present study, the reduced metabolic activity observed under breathing conditions, in the absence of marked inflammatory, oxidative, or fibrotic transcriptional responses, is therefore best interpreted as an early adaptive response to mechanical stimulation rather than evidence of overt cellular damage.

This interpretation is further supported by the secretory and stress-related readouts. EGFR expression showed only a modest increase under breathing conditions, while MUC5AC and MUC5B tended to be slightly reduced. Likewise, IL8 expression was significantly decreased, whereas TGFB and HMOX1 remained largely unchanged. Several studies have reported induction of IL8 in A549 cells under mechanical strain *via* the Cyr61/NF-κB pathway (Guo et al., 2009; Yang et al., 2018; Wang et al., 2020; Huang et al., 2021), but available literature also indicates that this response is strongly dependent on strain amplitude and exposure conditions, with little or no induction observed under more moderate mechanical stimulation (Ning and Wang, 2007a; Karadottir et al., 2015; Oudin and Pugin, 2002; Ning and Wang, 2007b; Yamamoto et al., 2002; Vlahakis et al., 1999). The downregulation of IL8 observed here may therefore reflect a homeostatic or protective adaptation to low-magnitude cyclic stretch, consistent with reports of mechanical preconditioning, in which physiological stretch attenuates inflammatory signalling compared with pathological loading (Fang et al., 2018). The use of soft fibrous membranes may have further contributed to this muted response, as substrate stiffness is known to modulate inflammatory signalling and mechanotransduction (Hess et al., 2009; Williams et al., 2021). The absence of substantial changes in TGFB and HMOX1 additionally suggests that the chosen strain amplitude and duration were insufficient to trigger marked pro-fibrotic or oxidative stress pathways. Since these markers are often associated with later or more pronounced stress responses, the present findings are most consistent with early biomechanical adaptation rather than overt stress induction (Yamamoto et al., 2002).

A similar interpretation applies to the aerosol exposure experiments. Under static ALI conditions, none of the tested aerosolised DQ12 doses measurably impaired viability, which is in agreement with previous ALI studies reporting limited cytotoxicity in epithelial mono- or co-cultures exposed to DQ12 in the range of approximately 0.2–10 μg/cm^2^ (Braakhuis et al., 2023; Meldrum et al., 2022). Based on these findings, a deposited dose of 0.31 μg/cm^2^ was selected for the DALI exposure experiments. This dose falls within the occupationally extrapolated *in vitro* dose window of 0–1 μg/cm^2^ established for DQ12 at the ALI using the MPPD dosimetry framework (Gangwal et al., 2011; PATROLS, 2025e), and was chosen to permit investigation of early pathway-specific responses rather than overload-associated toxicity. In this context, the absence of a measurable viability loss under breathing conditions is not unexpected and supports the suitability of the system for studying subtle epithelial responses under low-dose aerosol exposure.

At the transcriptional level, DQ12 exposure under breathing conditions induced only selective changes. CDH1 remained unchanged, PXN tended to decrease, and PTK2 showed a non-significant increase. The observed reduction in PXN may reflect early focal adhesion remodelling in response to particle-induced stress, whereas PTK2 upregulation may indicate compensatory signalling related to survival, cytoskeletal adaptation, or mechanotransduction (Grandy et al., 2023; Tomaru and Matsuoka, 2011; Mitra et al., 2005). Likewise, DQ12 did not significantly alter MUC5AC, MUC5B, or EGFR expression, which is consistent with previous observations that mucin-related pathways in epithelial models are not robustly induced by particulate exposure alone unless accompanied by additional cytokine stimulation (Rose and Voynow, 2006; Sousa de Almeida et al., 2023). By contrast, IL8 was upregulated after DQ12 exposure, consistent with a rapid early pro-inflammatory response previously described for silica at sub-cytotoxic concentrations (Monteiller et al., 2007). Since IL8 is regulated by NF-κB and can be influenced by both particle surface reactivity and mechanical stimulation, this finding suggests that low-dose aerosolised silica was sufficient to trigger a selective inflammatory transcriptional response under dynamic conditions (Churg et al., 2005; Tsuda et al., 1999; Ovrevik et al., 2004). In contrast, TGFB remained unchanged and HMOX1 did not increase despite the known ROS-generating potential of DQ12. For TGFB, this likely reflects the early observation window and the relatively low dose, as pro-fibrotic signalling is more commonly observed after stronger or more prolonged stimulation (Sun et al., 2025; Zhang et al., 2021; Barosova et al., 2020). The lack of HMOX1 upregulation may likewise indicate that the threshold for oxidative stress pathway activation was not reached under these low-dose ALI exposure conditions (Monteiller et al., 2007). Soft fibrous substrates have also been reported to attenuate mechanotransduction pathways linked to stress signalling, which may have further contributed to the limited oxidative response (Tschumperlin et al., 2014).

We should underline that although A549 cells are widely used in inhalation toxicology and represent a well-established model for proof-of-concept device validation, consistent with the approach taken in seminal lung-on-chip studies (Nikolic et al., 2018), their cancer-derived origin imposes some limitations. A549 cells do not form functional tight junctions, producing transepithelial electrical resistance values of 50–100 Ω cm^2^ that fall well below the threshold for a functional epithelial barrier (Ren et al., 2016; Cooney and Hickey, 2011), however, they can be cultured at the air-liquid interface and produce surfactant that lowers surface tension to values similar to those reported *in vivo* (Blank et al., 2006). Moreover, their mechanosensing and inflammatory signalling machinery may differ from that of non-transformed alveolar epithelium, which likely contributes to the generally muted transcriptional responses observed under both cyclic strain and DQ12 exposure in this study (Kryvenko and Vadasz, 2024). These are limitations of the biological model rather than of the DALI platform, which is architecturally compatible with more representative cell systems. The use of primary human alveolar epithelial cells, iPSC-derived alveolar type II cells, or co-culture models incorporating macrophages and endothelial cells represents a logical and necessary next step for application-oriented inhalation studies using DALI. The transition to such advanced cell models will, however, necessitate a more thorough assessment of epithelial barrier properties, which was beyond the scope of the current study. Incorporating impedance-based TEER monitoring and paracellular permeability assays into future iterations of the DALI platform would substantially strengthen its biological validity and broaden its applicability for physiologically relevant inhalation toxicology studies.

Taken together, the biological readouts observed in this study indicate a generally low but selective responsiveness of the epithelial model under the applied conditions, with a specific inflammatory response to aerosolised crystalline quartz (DQ12) in the absence of broad cytotoxic or stress-related perturbations. From an engineering perspective, these findings support the utility of the DALI system as a milli-scale platform that integrates biomimetic scaffolds, basal perfusion, cyclic stretch, air–liquid interface aerosol exposure, and dosimetry within an accessible and experimentally compatible format. By enabling the controlled combination of mechanical actuation and physiologically relevant aerosol delivery, DALI addresses key limitations of existing *in vitro* lung models, in which trade-offs between mechanical complexity, exposure realism, and quantitative dosimetry remain unresolved. The platform therefore provides a robust basis for controlled investigation of particle–epithelium interactions under dynamic conditions and represents a valuable tool for inhalation research and the development of physiologically relevant *in vitro* testing strategies.

## Data Availability

The raw data supporting the conclusions of this article will be made available by the authors, without undue reservation.
