# Supplementary material for: Impact of physiological strain on lung epithelial cells by exposure to aerosolised quartz silica in a perfused bioreactor
Source: Front Bioeng Biotechnol. 2026 Jun 15;14:1846147. doi: 10.3389/fbioe.2026.1846147 (PMC13311069; doi:10.3389/fbioe.2026.1846147)
Supplement: Supplementary file 1 [file DataSheet1.docx]

Impact of physiological strain on lung epithelial cells by exposure to aerosolised quartz silica in a perfused bioreactor

Ludovica Cacopardo^1*^, Nathalie Jung^2*^, Nicole Guazzelli^1,3°^, Roberta Nossa^4°^, Sandeep Keshavan^2^, Mira Witzig^2^, Alain Rohrbasser^2^, Mauro Sousa de Almeida^2^, Alke Petri-Fink^2^, Arti Ahluwalia^1+^, and Barbara Rothen-Rutishauser^2+^

^1^Department of Information Engineering and Research Centre E. Piaggio, University of Pisa, Largo Lucio Lazzarino 1, Pisa 56100, Italy

^2^BioNanomaterials group, Adolphe Merkle Institute, University of Fribourg, Ch. des Verdiers 4, 1700 Fribourg, Switzerland

^3^RANDSTAD DIGITAL ITALY S.r.l., PBPK M&S consulting department, Milano, Italy

^4^ItalyScientific Institute, IRCCS Eugenio Medea, Bosisio Parini (LC), Italy

*Contributed equally

^+^Corresponding authors:

Barbara Rothen-Rutishauser, Email: barbara.rothen@unifr.ch

Arti Ahluwalia, Email: arti.ahluwalia@unipi.it

°The contribution of these authors was carried out during their PhD studies at the Department of Information Engineering and the Research Centre E. Piaggio, University of Pisa.

**Supplementary Information**

**Design, fabrication, and testing of the DALI bioreactor**

The DALI bioreactor is composed of two main cylindrical chambers: an apical chamber (Figure S1D) that replicates the airside of the alveolus, and a basolateral chamber that mimics the vascular compartment. Both chambers were fabricated from medical-grade polycarbonate (PC) using subtractive manufacturing techniques, chosen for its optical transparency, mechanical durability, and compatibility with sterilization protocols. The apical chamber connects directly to a commercial nebulizer (Aeroneb Pro, Aerogen, Ireland) via a moulded polydimethylsiloxane (PDMS) collector, ensuring airtight integration for homogeneous aerosol delivery. The basolateral chamber is part of a closed-loop hydraulic circuit that allows continuous flow of cell culture medium, supported by a peristaltic pump (Ismatec IPC 4 ), and a modified PPCO reservoir (Nalgene™ - – figure 5 in the main manuscript) equipped with inlet, outlet, and filter tubes sealed in PDMS. Silicone tubing connects the entire circuit, preserving sterility and biocompatibility.

To replicate the mechanical stretch experienced by alveolar cells during breathing, the system incorporates an electropneumatic actuation module controlled by a custom-made Arduino-based control unit. Electropneumatic regulators (ITV0011-2BL, SMC) modulate cyclic pressurization of the apical chamber to generate linear strain ranging from physiological levels (5–12%) to pathophysiological conditions (>17%) at a breathing-relevant frequency of 0.2 Hz^1^. Details of the air circuit are reported in Figure S1. The Arduino Micro board regulates the actuation signal through pulse-width modulation (PWM), which is converted to analog voltage by an integrated low-pass RC filter. Stretching parameters, including pressure levels and strain settings, are displayed in real time on an LCD interface to facilitate operator control (Figure 1S E). Parallel operation of two bioreactors is enabled by dual-channel regulation, allowing independent modulation of airflow and perfusion while preventing cross-contamination.


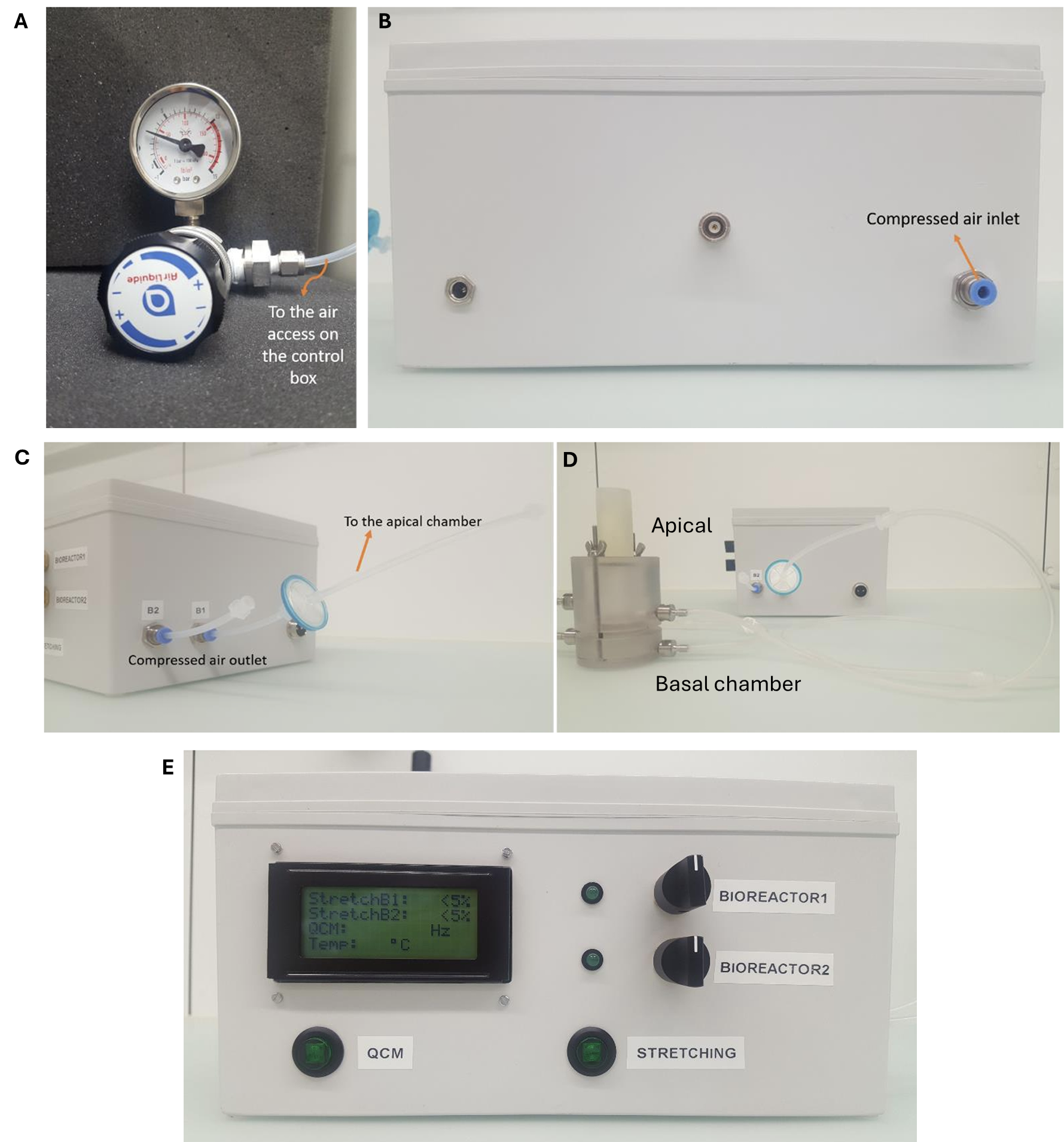


*Figure S1.* ***A/B****: Co/nnection to external air pressure supply: coupling with the Aeroneb nebulizer,* ***C/D****: air delivery to apical chamber,* ***E****: control box user interface*

An elastic membrane is positioned between the two chambers using a specially designed holder composed of neodymium magnets fully coated in PDMS, providing secure fixation without mechanical damage. This design ensures membrane flatness and tension during dynamic stimulation, while preserving cytocompatibility and allowing sterilization with ethanol or UV light.

To precisely quantify the dose of aerosolized nanoparticles delivered to the cell layer seeded on the membrane, a custom Quartz Crystal Microbalance (QCM) was developed. This device integrates a 25.4 mm AT-cut quartz crystal mounted on spring contacts within a 3D-printed PLA support, replicating the geometry and exposure conditions of the DALI bioreactor. The same apical chamber is used for both QCM and DALI setups, ensuring that deposition occurs under equivalent conditions. Figure S2 shows details of the construction and assembly of the QCM module . The resonant frequency shift of the QCM crystal is detected using a commercial controller (QCM200, Stanford Research Systems) and converted into mass via the Sauerbrey equation:

$$@\Delta m=\frac{\Delta f\cdot A}{-2.26\cdot{10}^{-6}\cdot n\cdot f_{0}^{2}}$$

An embedded temperature sensor compensates for thermal drift, and the QCM can also be connected directly to the DALI control unit, where a crystal oscillator driver (SN74LVC1GX04, Texas Instruments) and I2C-linked temperature sensor enable direct signal acquisition.


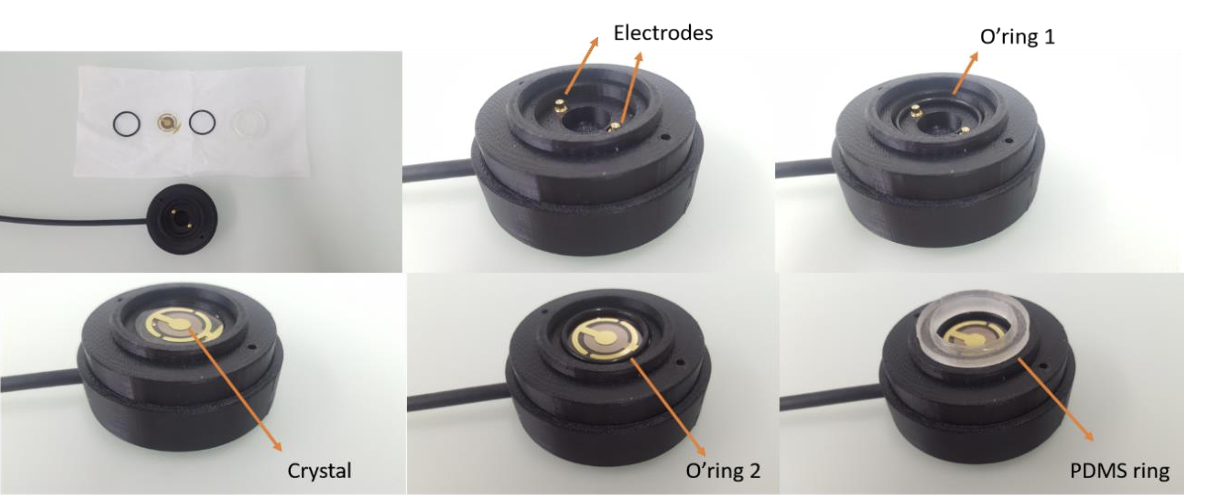


Figure S2. QCM module, showing in sequence: the different components and their assembly. Spring electrodes ensure electric connection between the quartz crystal and the control box circuit.

**Additional data on mRNA expression of EGFR and HMOX1**

Exploratory qRT-PCR data for mRNA expression of EGFR and HMOX1 (n = 2).


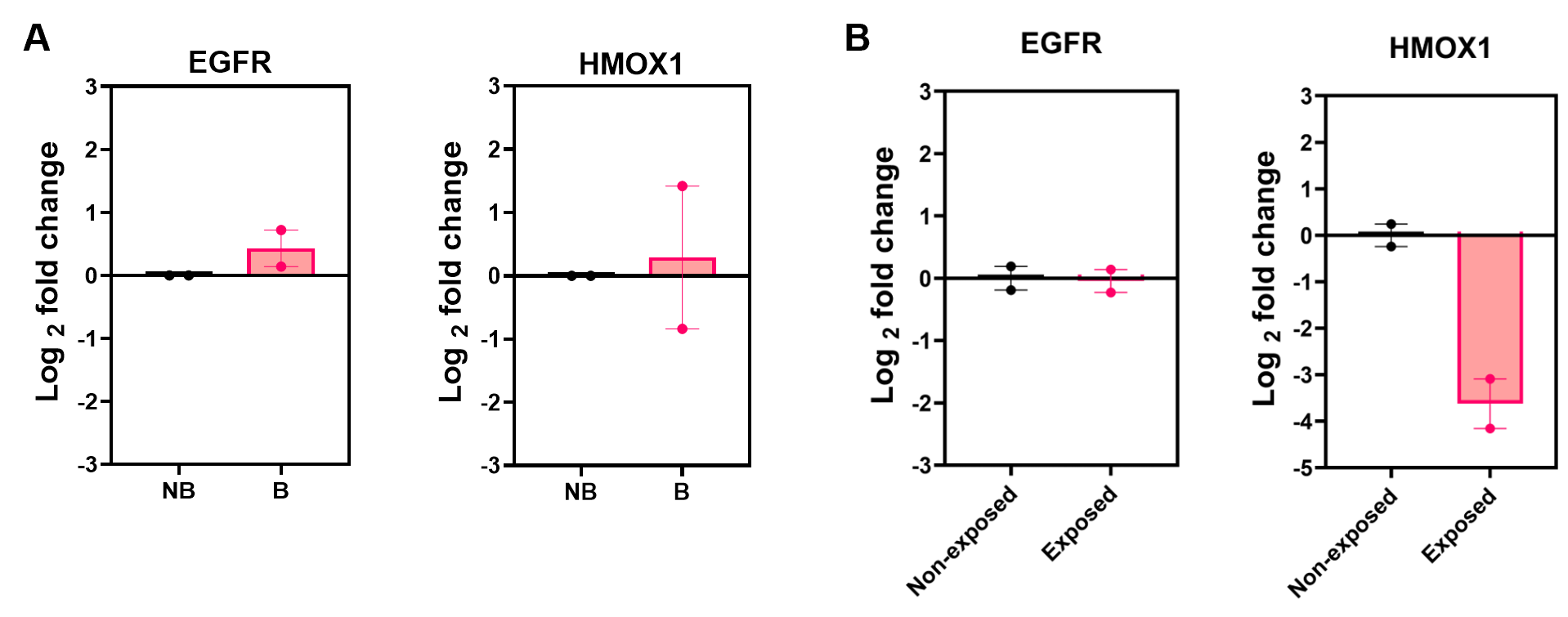


Figure S3. Exploratory qRT-PCR analysis of EGFR and HMOX1 expression. **A**: mRNA expression of epidermal growth factor receptor (EGFR) and heme oxygenase 1 (HMOX1) under NB and B conditions. **B**: mRNA expression in breathing models under non-exposed conditions and following exposure to 0.31 µg/cm² deposited DQ12 (5% cyclic stretch, 0.2 Hz, 6 h). Data are normalised to YWHAZ and presented as mean ± SD (n = 2 biological replicates). No statistical analysis was performed.

**Comparative mRNA expression analysis of NB and B A549 tissues after DQ_12_ exposure**

The exposure to aerosolised DQ_12_ in the selected concentration (10 μL of 2.56 mg/mL solution, resulting in 0.31μg/cm^2^ deposited DQ_12_) did not lead to a change in mRNA expression for the selected genes depending on the culture condition (NB vs. B) in A549 cultures (Figure S4).


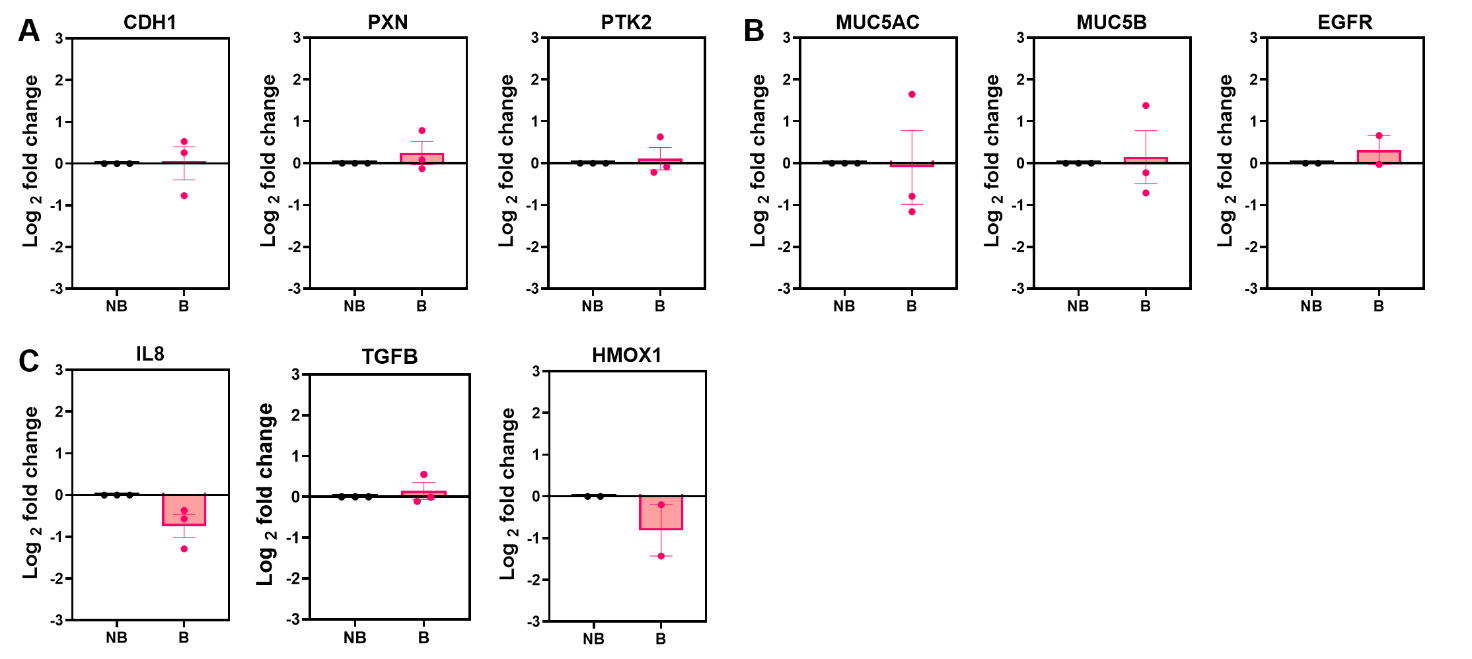
*Figure S4:* Comparison of mRNA expression between NB and B conditions after DQ_12_ exposure (*10 μL of 2.56 mg/mL solution were aerosolised on A549 cultures, resulting in 0.31μg/cm^2^* deposited *DQ_12_, B conditions: 5%* *cyclic* *stretch,* *0.2 Hz,* *6 h),* *assessed* *by* *WST-1* *assay.* **A***:* *mRNA* *expression* *of* *cell* *junction* *and* *focal* *adhesion* *markers:* *E-cadherin* *(CDH1),* *focal* *adhesion* *kinase* *(PTK2),* *and* *paxillin* *(PXN) for B-condition.* **B***:* *mRNA* *expression* *of* *mucin-associated* *genes* *and* *regulators:* *mucin* *5AC* *(MUC5AC),* *mucin* *5B* *(MUC5B),* *and* *epidermal* *growth* *factor* *receptor* *(EGFR) for B-condition.* **C***:* *mRNA* *expression* *of* *stress* *response* *markers:* *interleukin-8* *(IL8),* *transforming* *growth* *factor* *beta* *(TGFB),* *and* *heme* *oxygenase* *1* *(HMOX1) for B-condition.*

Table S1: Primers for qRT-PCR.

| Gene of Interest | Primer Sequence (5′ → 3′) | Concentration [nM] | Primer Efficiency [%] |
| --- | --- | --- | --- |
| YWHAZ | FW: GCTGGTGATGACAAGAAAGGGAT | 91 | 72.80 |
|  | RV: GTTAAGGGCCAGACCCAGTC |  |  |
| IL8 | FW: GAGAAGTTTTTGAAGAGGGCTGA | 91 | 75.69 |
|  | RV: GCTTGAAGTTTCACTGGCATCT |  |  |
| TGFβ | FW: CCCTACATTTGGAGCCTGGACACG | 182 | 83.75 |
|  | RV: CGGGTTATGCTGGTTGTACAGGGC |  |  |
| MUC5AC | FW: AGCTATGTGCTGACCAAGCC | 182 | 81.19 |
|  | RV: TGATCACCACCACCGTCTGC |  |  |
| MUC5B | FW: CGTTCTGTCCAAGAAATGTGC | 91 | 84.11 |
|  | RV: AGTTCTCGTTGTCCGTCAGG |  |  |
| CDH1 | FW: CTGGACCGAGAGAGTTTCCC | 91 | 87.52 |
|  | RV: CGTGGTGGGATTGAAGATCG |  |  |
| PTK2 | FW: CAATCCCACACATCTTGCTGA | 91 | 80.42 |
|  | RV: AGCCGGCAGTACCCATCTATT |  |  |
| PXN | FW: AACTTCTTCGAGCGGGATGG | 91 | 118.53 |
|  | RV: AGGGCTGTCACCACTTTATCC |  |  |

**References**

1. Nossa R, Costa J, Cacopardo L, Ahluwalia A. Breathing in vitro: Designs and applications of engineered lung models. *J Tissue Eng*. Jan-Dec 2021;12:20417314211008696. doi:10.1177/20417314211008696
